# Bruton tyrosine kinase inhibitors and invasive aspergillosis and mucormycosis: a case report and multi-center exploratory retrospective study

**DOI:** 10.1128/asmcr.00087-25

**Published:** 2025-11-26

**Authors:** Eric Bhaimia, Daniel Russell, Carlos A. Q. Santos

**Affiliations:** 1 Division of Infectious Diseases, Department of Internal Medicine, Rush University Medical Center 2468, Chicago, Illinois, USA; 2 Division of Pathology, Rush University Medical Center 2468, Chicago, Illinois, USA; Vanderbilt University Medical Center, Nashville, Tennessee, USA

**Keywords:** *Aspergillus*, mucor, bruton tyrosine kinase inhibitors, invasive fungal infection

## Abstract

**Background:**

Bruton tyrosine kinase inhibitors (BTKIs) are increasingly utilized for patients with certain malignant hematologic conditions. While cases of invasive fungal infections have been reported in patients on BTKIs, no multi-center epidemiological studies have been performed. The Epic Cosmos platform, a nationwide electronic health record data set, provides an opportunity to assess real-world epidemiologic data.

**Case Summary:**

A 69-year-old female with recently diagnosed Waldenström macroglobulinemia with associated autoimmune hemolytic anemia undergoing treatment with zanubrutinib and prednisone presented with fatigue and acute kidney injury. Imaging demonstrated a bladder mass with bilateral hydronephrosis leading to transurethral resection of the bladder mass with histopathology revealing both broad ribbon-like hyphae resembling Mucorales spp. and acute-angle branching resembling *Aspergillus* spp. with angioinvasion. Additional imaging revealed bilateral pulmonary cavitary lesions and renal and brain involvement. Bronchoalveolar lavage returned a positive galactomannan and culture growth of *Aspergillus fumigatus*. The patient was treated with liposomal amphotericin B with eventual transition to oral isavuconazole. Zanubrutinib and steroids were discontinued. To assess real-world occurrence of IFIs in patients on BTKIs, we performed an exploratory analysis using Epic Cosmos.

**Conclusion:**

IFIs continue to be described in patients undergoing treatment with BTKIs. Through exploratory analysis using Epic Cosmos, aspergillosis occurred not infrequently (0.5%–1.9%) in patients on various BTKIs. Of patients on ibrutinib with a diagnosis of chronic graft-versus-host disease, aspergillosis was identified in 32 (8.3%) patients. Patients on BTKIs who developed aspergillosis were frequently on corticosteroids. Mucormycosis in patients on BTKIs was rare (<0.1%).

## INTRODUCTION

Bruton tyrosine kinase inhibitors (BTKIs) are used for the treatment of several malignant hematological conditions, including chronic lymphocytic leukemia/small lymphocytic lymphoma (CLL/SLL) (1–4), previously treated Waldenström macroglobulinemia (5, 6), relapsed/refractory follicular lymphoma (7), relapsed/refractory mantle cell lymphoma (8–10), and relapsed/refractory marginal zone lymphoma (11). In addition, the BTKI, ibrutinib, is used for treatment of chronic graft-versus-host disease (GVHD) after failure of one or more lines of systemic therapy in allogeneic hematopoietic cell transplant (HCT) recipients (12). All BTKIs cause immunocompromise by inducing B-cell dysfunction and decreasing serum immunoglobulin levels (13, 14). Moreover, ibrutinib also inhibits interleukin-2-inducible T-cell kinase, which reduces T-cell activation (15).

A few instances of invasive fungal infections (IFIs) have been identified in clinical trials on ibrutinib for CLL (2, 4, 6, 8, 12). Case reports and case series have described aspergillosis in patients on various BTKIs (16–18) and mucormycosis in patients on ibrutinib and zanubrutinib (19, 20). The risk of IFI is variable based on disease status and prior treatment regimens. As BTKIs are increasingly used in clinical practice, there is a need to determine the epidemiology of IFIs in these patients in real-world settings as part of pharmacovigilance efforts in detecting adverse events.

Herein, we review a patient on zanubrutinib who developed both aspergillosis and mucormycosis, the first report of dual infection with these molds in the literature and present the results of an exploratory multi-center retrospective study of patients on BTKIs who subsequently developed aspergillosis or mucormycosis using data from Epic Cosmos. Understanding the epidemiology of aspergillosis and mucormycosis in patients on BTKI therapy may inform prevention strategies.

## CASE PRESENTATION

The patient is a 69-year-old female with autoimmune hemolytic anemia on prednisone 60 mg twice daily 6 weeks prior to admission, subsequently diagnosed with Waldenström macroglobulinemia and started on concurrent zanubrutinib 4 weeks prior to admission, who was transferred to our institution for evaluation of a urinary bladder mass. Upon initial presentation to another hospital, she noted generalized fatigue and was found to have acute kidney failure, a urinary bladder mass, and bilateral hydronephrosis for which she underwent transurethral resection of bladder tumor, bilateral ureteral stent placement, and hemodialysis. Preliminary histopathology of the bladder mass showed fungal hyphae consistent with angioinvasive mold for which she was started on intravenous liposomal amphotericin B (LAmB) 5 mg/kg and transferred to our institution.

**Fig 1:**
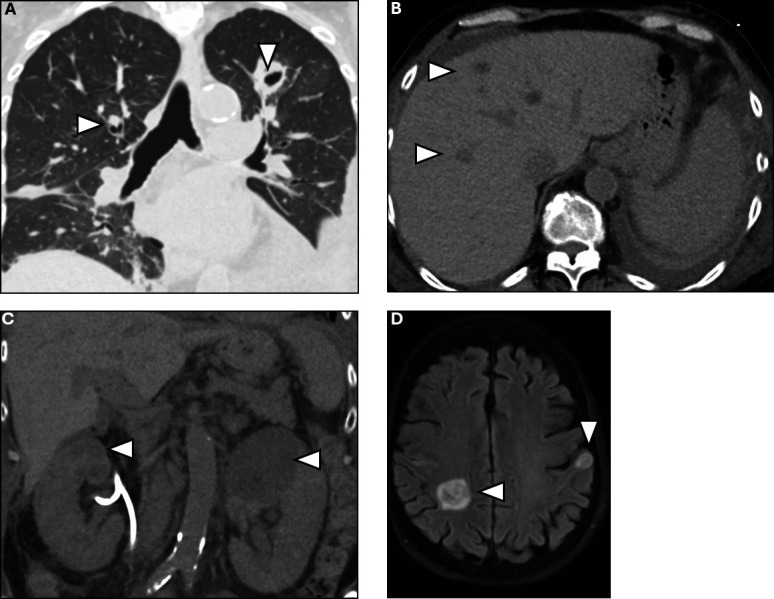
Non-contrast CT of the chest, abdomen, and pelvis demonstrating bilateral cavitary nodules (**A**), hepatic hypodensities (**B**), and bilateral kidney hypodensities (**C**) suggestive of abscesses. T2 FLAIR MRI brain with and without contrast demonstrating right parietal lobe and left temporal lobe hyperintensities consistent with septic emboli (**D**).

She reported several weeks of fatigue and decreased urine output prior to presentation. She lives in the Midwestern United States without a history of travel. She was afebrile on admission with stable vital signs. Physical exam was notable for coarse breath sounds bilaterally and bilateral lower extremity pitting edema. Labs were notable for a white blood cell count of 22.9 K/µL (normal range 4–10 K/µL) with 94.9% neutrophils, hemoglobin 9.8 g/dL (normal range 12–16 g/dL), platelets 88 K/µL (normal range 150–399 K/µL), blood urea nitrogen 48 mg/dL (normal range 8–21 mg/dL), and serum creatinine 2.8 mg/dL (normal range 0.6–1.0 mg/dL), and liver and coagulation studies were normal. A computed tomography (CT) scan of the chest, abdomen, and pelvis without intravenous (IV) contrast showed multiple bilateral cavitary lung nodules (Fig. 1A), multiple hypodensities in the liver (Fig. 1B), and bilateral hypodense kidney lesions concerning for abscesses (Fig. 1C). A magnetic resonance imaging (MRI) scan of the brain without and with IV gadolinium showed multiple areas of restricted diffusion with associated subtle enhancement concerning for septic emboli (Fig. 1D). Given these findings, the patient was assessed to have disseminated angioinvasive mold infection related to zanubrutinib and prednisone, and LAmB was continued. Zanubrutinib and prednisone were discontinued.

**Fig 2:**
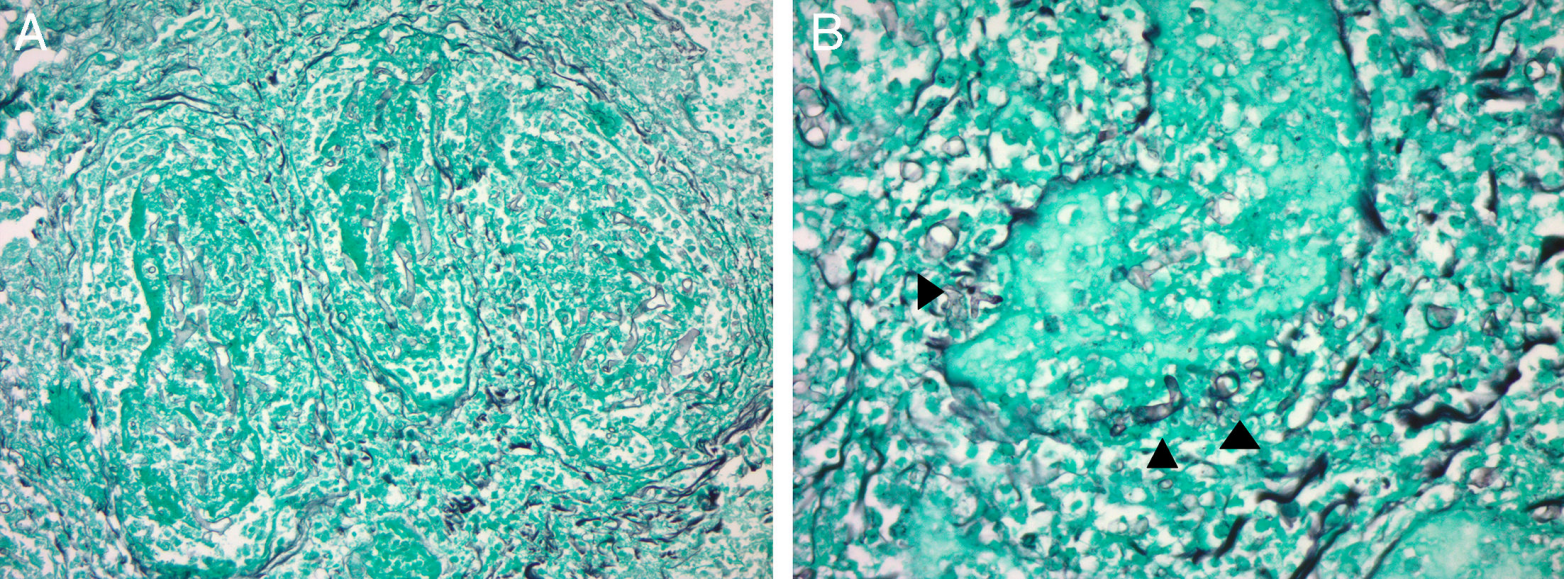
Angioinvasive fungal elements comprised of aseptate broad ribbon-like hyphae with wide angle branching, as seen in *Mucormycosis* spp., with perforation of the vessel wall (arrowheads) (**A**: 20× magnification, Grocott-Gomori methenamine silver stain; **B**: 40× magnification, Grocott-Gomori methenamine silver stain).

**Fig 3:**
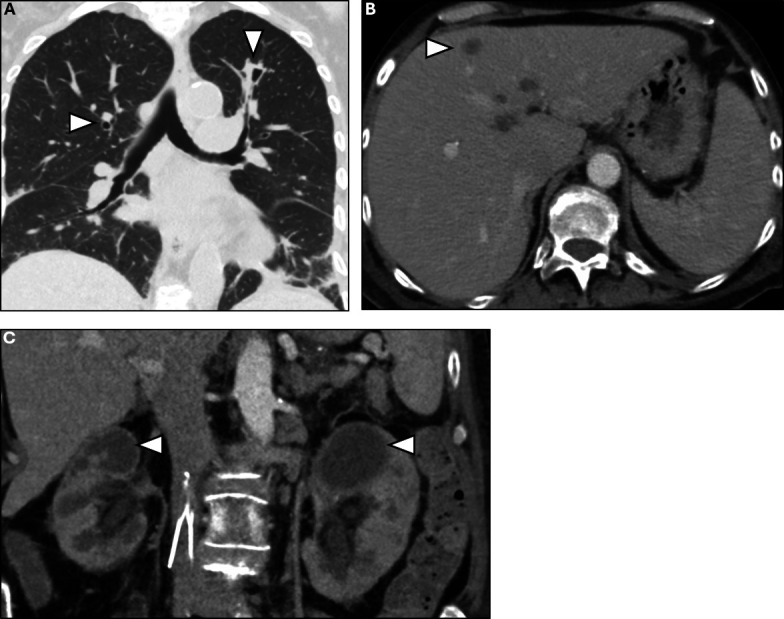
Non-contrast CT of the chest demonstrating reduction in bilateral cavitary infiltrates (**A**). Contrast CT of the abdomen and pelvis demonstrating stable hypodensities in the liver and kidneys (**B and C**).

The patient underwent bronchoscopy with bronchoalveolar lavage (BAL), revealing rare growth of *Aspergillus fumigatus* on fungal culture. A BAL galactomannan antigen was positive with an optical density index of 5.1 (normal value ≤0.5), and a serum galactomannan was negative. Fungal culture from urine at the referring hospital showed Zygomycetes. Histopathology of the bladder mass showed invasive fungal elements with extensive angioinvasion (Fig. 2). Grocott’s methenamine silver stain revealed morphological heterogeneity of fungal elements, with both broad ribbon-like structures with wide-angle branching typically seen with Zygomycetes and narrow hyphae with acute-angle branching typically seen with *Aspergillus* species (Fig. 2). Further speciation of the zygomycete was attempted in coordination with the referring hospital but was unable to be performed. Disseminated zygomycete infection was presumed based on isolation from a non-pulmonary site, and disseminated *Aspergillus* infection was confirmed by isolation from the urinary bladder and lungs. Therapy with LAmB was continued for 6 weeks with eventual transition to oral isavuconazole. The patient improved steadily and was ambulatory at the time of LAmB discontinuation. A CT scan of the chest, abdomen, and pelvis 5 weeks after antifungal therapy showed significant reduction in size of the multifocal consolidations and nodular opacities (Fig. 3A), stable multiple hypodense hepatic lesions (Fig. 3B), and persistent bilateral hypodense kidney lesions (Fig. 3C). An MRI of the brain had not yet been repeated. She was discharged home on isavuconazole with plans for close follow-up in the outpatient setting.

## DISCUSSION

This case of disseminated dual infection with *A. fumigatus* and Zygomycetes in a patient on zanubrutinib and steroids who was not receiving IFI prophylaxis prompted us to perform an exploratory, multi-center retrospective study of patients on BTKIs who subsequently developed aspergillosis or mucormycosis using data from Epic Cosmos, an integrated EHR database with information on more than 270 million patients (as of the study period) from all 50 states in the United States (21). We defined our study period as 10 October 2021 to 9 October 2024 to capture a more recent patient cohort. The database was accessed using the Cosmos data exploration tool. The study was deemed exempt by the RUSH University Institutional Review Board.

We identified patients on BTKIs separately and determined if they were diagnosed with aspergillosis or mucormycosis after initiation of BTKIs. BTKIs were identified using medication names; aspergillosis and mucormycosis were identified using ICD-10 diagnosis codes (Table 1). Patients on BTKIs who subsequently developed aspergillosis and mucormycosis were characterized in terms of gender, race, age, receipt of corticosteroids, and vital status. Corticosteroids were identified using a medication grouper in Epic Cosmos (21). In separate analyses, we identified labeled indications for each BTKI and analyzed the percentage of patients with a new diagnosis of aspergillosis and mucormycosis (new IFI) by labeled indication using ICD-10 diagnosis codes (Table 2). Cell sizes with <10 patients were obscured to maintain deidentification. All descriptive analyses were done on Epic Cosmos since no data can be exported from the platform.

**TABLE 1:** ICD-10 diagnosis codes used for data extraction

Condition	ICD-10 diagnosis codes
Aspergillosis	B44.9, B44.2, B44.7, B44.89, B44.1, B44.0
Mucormycosis	B46.5, B46.3, B46.0, B46.4, B46.1, B46.2
Chronic GVHD	D89.811
CLL, SLL	C91.1, C91.12, C91.11, C91.10; C83.0, C83.07, C83.00, C83.06, C83.02, C83.03, C83.08, C83.09, C83.01, C83.04, C83.05
Waldenström macroglobulinemia	C88.0
Follicular lymphoma	C82.0, C82.1, C82.3, C82.4, C82.07, C82.17, C82.37, C82.47, C82.2, C82.00, C82.10, C82.30, C82.40, C82.27, C82.06, C82.02, C82.16, C82.03, C82.13, C82.12, C82.36, C82.32, C82.46, C82.42, C82.08, C82.18, C82.33, C82.43, C82.09, C82.19, C82.20, C82.38, C82.48, C82.04, C82.01, C82.11, C82.39, C82.49, C82.14, C82.26, C82.34, C82.31, C82.44, C82.41, C82.23, C82.22, C82.05, C82.15, C82.28, C82.29, C82.35, C92.45, C82.24, C82.21, C82.25, C82.9, C82.96, C82.92, C82.93, C82.98, C82.99, C82.91, C82.95, C82.8, C82.87, C82.80, C82.86, C82.82, C82.83, C82.88, C82.89, C82.81, C82.84, C82.85
Mantle cell lymphoma	C83.1, C83.17, C83.10, C83.16, C83.12, C83.13, C83.18, C83.19, C83.14, C83.11, C83.15
Marginal zone lymphoma	C88.4, C83.07

**TABLE 2:** Percentage and characteristics of patients with aspergillosis and mucormycosis in patients on BTKIs

Variable	Ibrutinib (*N* = 27,772)	Zanubrutinib (*N* = 11,352)	Acalabrutinib (*N* = 16,933)	Pirtobrutinib (*N* = 1,308)
Aspergillosis, no. (%)	265 (1.0)	61 (0.5)	121 (0.7)	25 (1.9)
Female	31 (11.6)	22 (36.1)	32 (26.4)	≤10
White	223 (84.1)	53 (86.9)	109 (90.1)	24 (96.0)
≥65 years	188 (70.9)	45 (73.8)	92 (76.0)	19 (76.0)
Corticosteroids	195 (73.6)	47 (77.0)	109 (90.1)	22 (88.0)
Death	75 (28.3)	20 (32.8)	43 (35.5)	13 (52.0)
Mucormycosis, no. (%)	19 (0.1)	≤10	≤10	≤10

Patient characteristics and percentage experiencing new IFI with recorded use of BTKIs are listed in Table 2. Patients on ibrutinib were most frequently identified. The incidence of aspergillosis ranged from 0.5% in patients on zanubrutinib to 1.9% in patients on pirtobrutinib. Patients with aspergillosis on BTKIs were predominantly male, white, >65 years of age, and on corticosteroids. The occurrence of death during the study period ranged from 28.3% of patients with aspergillosis on ibrutinib to 52.0% of patients with aspergillosis on pirtobrutinib. Mucormycosis was rare and could only be reported for patients on ibrutinib of whom 0.1% were identified to have mucormycosis.

The percentage of aspergillosis and mucormycosis in patients on BTKIs stratified by indication for BTKI treatment is detailed in Table 3. The majority of patients on BTKIs had a diagnosis of CLL/SLL in whom the incidence of aspergillosis ranged from 0.5% on zanubrutinib to 2.1% on pirtobrutinib. Among patients on ibrutinib who had a diagnosis of chronic GVHD, the incidence of aspergillosis was 8.3%. Mucormycosis was rare and not reportable on account of small cell sizes, defined as less than 10 occurrences.

**TABLE 3:** Labeled indications for BTKIs and percentage of new aspergillosis and mucormycosis

Variable	Ibrutinib (*N* = 27,772)	Zanubrutinib (*N* = 11,352)	Acalabrutinib (*N* = 16,933)	Pirtobrutinib (*N* = 1,308)
Chronic GVHD, no. (%)	387 (1.4)	NA^*a*^	NA	NA
Aspergillosis	32 (8.3)			
Mucormycosis	≤10			
CLL/SLL, no. (%)	19,206 (69.2)	7,395 (65.1)	13,831 (81.7)	816 (62.4)
Aspergillosis	167 (0.9)	38 (0.5)	92 (0.7)	17 (2.1)
Mucormycosis	≤10	≤10	≤10	≤10
Waldenström macroglobulinemia, no. (%)	2,821 (10.2)	2,610 (23.0)	NA	NA
Aspergillosis	21 (0.7)	≤10		
Mucormycosis	≤10	≤10		
Follicular lymphoma, no. (%)	NA	446 (3.9)	NA	NA
Aspergillosis		≤10		
Mucormycosis		≤10		
Mantle cell lymphoma, no. (%)	NA	1,614 (14.2)	1,928 (11.4)	508 (38.8)
Aspergillosis		11 (0.7)	18 (0.9)	≤10
Mucormycosis		≤10	≤10	≤10
Marginal zone lymphoma, no. (%)	NA	820 (7.2)	NA	NA
Aspergillosis		≤10		
Mucormycosis		≤10		

^*a*^ NA indicates not applicable.

Our findings suggest that factors leading to a high net state of immunosuppression contribute to the development of aspergillosis in patients on BTKIs. Aspergillosis and mucormycosis have high rates of morbidity and mortality in immunocompromised hosts (22, 23), and patients at highest risk have been shown to benefit from antifungal prophylaxis, specifically posaconazole (24). Posaconazole and voriconazole have class D interactions with ibrutinib, zanubrutinib, and pirtobrutinib and class X interactions with acalabrutinib, making the use of triazoles difficult in patients on BTKIs. Alternative agents in development with potential for prophylactic use include fosmanogepix, ibrexafungerp, and olorofim (25–27). Clinical trials with these agents in patients at high risk for IFIs may benefit patients on BTKIs.

Other factors leading to high net states of immunosuppression likely contribute to the development of aspergillosis and mucormycosis in patients on BTKIs. Like our patient, most patients with aspergillosis on BTKIs were on corticosteroids and presumably not on triazoles owing to drug interaction. Large-scale epidemiologic studies with access to patient-level information are needed to predict the occurrence of IFIs in patients on BTKIs as part of pharmacovigilance as well as directing preventive strategies (28). This may be possible with the Epic Cosmos data science virtual machine or other multi-center databases such as PCORnet or TriNetX (21, 29, 30).

Our study, while novel, has limitations, including use of ICD-10 diagnosis codes for identification of infection and BTKI indication, and inability to fully characterize patients with infection (31). Similarly, details surrounding precise timing of IFI diagnosis relative to BTKI administration were not available in the data exploration tool of Epic Cosmos, and we did not fully characterize other risk factors for the development of IFI such as neutropenia or those who subsequently received hematopoietic cell transplantation. However, our study provides preliminary data that informs the design of large-scale epidemiologic studies with patient-level data using the Epic Cosmos data science virtual machine or other multi-center databases (21, 29, 30). Other case reports of aspergillosis and mucormycosis in patients on BTKIs have already been reported, with outcomes of mucormycosis in immunocompromised hosts well characterized (16–20).

In summary, we encountered a case of dual disseminated infection with *A. fumigatus* and Zygomycetes in a patient on BTKI therapy and found in an exploratory multi-center study that aspergillosis occurred not infrequently in patients on BTKIs. The incidence of aspergillosis was greatest in patients on ibrutinib for chronic GVHD, followed by patients on pirtobrutinib for CLL/SLL. Mucormycosis in patients on BTKIs was rare. Our findings suggest that corticosteroid use and underlying relapsed/refractory malignant hematological disease are associated with the development of aspergillosis in patients on BTKIs.
